# Inter-disciplinary approaches for the infectious diseases pathology

**DOI:** 10.1186/1471-2334-14-S7-O23

**Published:** 2014-10-15

**Authors:** Bogdan Cîrciumaru, Carmen Sarbu, Daniela Badescu, Marius Balea, Vasile Murgu, Ionel Droc

**Affiliations:** 1 Central Universitary Emergency Military Hospital Dr Carol Davila, Bucharest, Romania; 2 Cantacuzino National Institute for Research and Development for Microbiology and Immunology, Bucharest, Romania; 3 Colentina Clinical Hospital, Bucharest, Romania; 4 Army's Center For Cardiovascular Diseases, Bucharest, Romania

## Background

In order to elucidate the diagnosis and the correct therapeutic approaches, the present high degree of specialization, as well as the complex, frequent “borderline” pathology, impose an inter-disciplinary cooperation. We presented the main infections that imposed an inter-disciplinary cooperation; we enunciate the main imposed problems, as well as a unitary vision for these approaches.

## Methods

We sorted out the cases that necessitated at least two admissions in different specialty wards, compulsory, one of them being the Central Universitary Emergency Military Hospital Dr Carol Davila Infectious Diseases Ward, for diagnosis, therapy and follow-up.

## Results

The most frequent inter-disciplinary approaches were: infections in immunosuppressed patients (HIV/AIDS, tumors, immune-suppressive therapies) with oncology and hematology; infectious endocarditis with cardiology and cardio-surgery; hydatid diseases with general surgery, infections in drug abusers with psychiatry; neurological pathology (e.g. neuroborreliosis) with neurology. In order to sustain the diagnosis, we used the hospitals’ exploring facilities, as well as national and international reference laboratories.

## Conclusion

The interdisciplinary approaches were the success key for diagnosing and treating patients, being a necessary tradition, due to the complexity of cases. We proposed optimal ways of collaboration, sometimes successfully, but institutionalized approaches protocols were needed.

